# Mitigating inflammation and fibrosis: the therapeutic potential of quercetin liposomes in COPD

**DOI:** 10.3389/fphar.2024.1503283

**Published:** 2024-12-17

**Authors:** Changfeng Yin, Yushan Tian, An Yan, Hongjuan Wang, Fengjun Lu, Xianmei Li, Xiao Li, Shulei Han, Ruijuan Miao, Huan Chen, Di Li, Hongwei Hou, Qingyuan Hu

**Affiliations:** ^1^ China National Tobacco Quality Supervision & Test Center, Zhengzhou, China; ^2^ Beijing Life Science Academy, Beijing, China; ^3^ Key Laboratory of Tobacco Biological Effects, Zhengzhou, China; ^**4**^ School of Chemistry and Molecular Engineering, East China Normal University, Shanghai, China

**Keywords:** chronic obstructive pulmonary disease, inflammation, fibrosis, quercetin, liposomes

## Abstract

**Introduction:**

Chronic obstructive pulmonary disease (COPD) is a disease with severe therapeutic obstacles and high worldwide death rate. COPD progresses predominantly through inflammatory response followed by fibrotic destruction. Quercetin (Que), recognized for its anti-inflammatory effects, presents significant promise as a therapeutic candidate for COPD therapy. However, poor water solubility and low bioavailability of Que hinder its further clinical application. Liposomes are renowned for their unique structure and function, which provided an efficient approach for the delivery of Que in various drug delivery systems. This study was aim to prepare a novel Que liposome (Que-lipo) and administrated via intratracheal (i.t.) with cigarette smoke induced COPD mice. The underlying therapeutic mechanisms against lung damage of Que-lipo were explored.

**Methods:**

Que-lipo were prepared based on thin film dispersion method and administrated via intratracheal administration. The cigarette smoke induced COPD mice were established and a comprehensive approach was employed to explore the inflammation, pulmonary function and histopathology of lung after i.t. administration of Que-lipo, including enzyme-linked immunosorbent assay, histopathology and immunohistochemistry, reverse transcription-quantitative polymerase chain reaction.

**Results and discussion:**

Que-lipo not only improved the solubility and biocompatibility of Que but also demonstrated effective cellular uptake *in vitro*. The inflammation, pulmonary function and pathological condition of lung were improved after i.t. administration of Que-lipo. Que-lipo also regulated the expression of key apoptosis-associated proteins such as Bcl-2 and caspase-3/7, leading to significant inhibition of apoptotic activity in COPD. Furthermore, Que-lipo markedly enhanced its ability to alleviate lung inflammation and fibrosis symptoms by modulating inflammation-related factors and fibrosis signaling molecules. The potential mechanisms of Que-lipo in treating COPD were elucidated, including the suppression of the NLRP3/IL-1β inflammasome pathway and the TGF-β1-related fibrosis signaling pathway.

## 1 Introduction

Chronic obstructive pulmonary disease (COPD) is a chronic lung condition characterized by persistent and progressive airflow limitation, chronic inflammation and emphysematous destruction of the lungs (Stolz et al., 2022). According to the World Health Organization report in 2020, COPD has become the third leading cause of death globally (Li et al., 2022). With the increase of aging population, its incidence is gradually raised. Smoking is identified as the main risk factor of COPD (Araújo et al., 2022) evidenced by oxidative stress and inflammation (Guo et al., 2022; Han et al., 2020). This chronic inflammation in COPD involves in the number of cells, including macrophages, lymphocytes, and neutrophils in the pulmonary blood vessels, peripheral airways, and lung. These cells can release different inflammatory mediators interacted with other structural cells, leading to an exacerbation of the inflammatory response. Oxidative stress plays a key role in COPD related inflammation, as well as in smokers. In clinical, the primary pharmacological interventions for COPD include inhaled corticosteroids and β2 agonists (Terry and Dhand, 2020), which are reported to induce many adverse effects such as rapid heartbeat, metabolic disorders and infections (Billington et al., 2017; Liapikou et al., 2015). Therefore, the potential therapy approaches with better pharmacodynamics and relatively no side effects are urged for treatment of COPD. Furthermore, drugs with powerful anti-inflammatory and antioxidant ability are a potential candidate for COPD treatment.

Flavonoids were reported to exert strong antioxidant activity than the most compounds. Among them quercetin (Que) is one of the most powerful antioxidants in flavonoids and widely exists in many plants, known for its good anti-inflammatory property (Ghafouri-Fard et al., 2021; Grewal et al., 2021; Zhao et al., 2021). Numerous researches had shown that Que effectively reduced oxidative stress and inflammation mediated by decreasing reactive oxygen species levels, thus protected the lungs from lipopolysaccharide-induced injury (Sul and Ra, 2021), which indicated that Que may provide potential protective effects in the pathogenesis of COPD. It has been reported that lung damage and the production of pro-inflammatory cytokines were significantly reduced in mice after orally administered (20, 40, 60 mg/kg) and intraperitoneally (i.p., 50 mg/kg) treatment with Que (Chen L.-L. et al., 2022a; Deng et al., 2023). In smoking-related COPD model, airway inflammation and the progression of COPD were restored by Que (Ding et al., 2023). However, the low solubility and bioavailability have limited its application.

New treatment methods, particularly novel inhalation therapy, provide significant advantages over traditional drug delivery methods for COPD (Chen Y.-B. et al., 2022b). It can deliver the drugs directly to the location of the disease, enhancing its utilization by mitigating hepatic first-pass metabolism and circumventing gastrointestinal malabsorption, and reduce the adverse effects caused by oral or intravenous drug delivery methods (Zheng et al., 2024). Moreover, new techniques in inhalation therapy, including the utilization of liposomes and nanoparticles, have demonstrated efficacy in treating respiratory diseases. Innovative nano-drug delivery systems, such as alginate nanogels and chitosan-assisted nanoparticles, have been employed to increase its bioavailability and therapeutic efficacy of Que, offering new strategies for treating acute lung injury (Deng et al., 2023; Chen Y.-B. et al., 2022b). These findings indicated that the potential therapeutic of Que in treating pulmonary diseases. In consideration of the hydrophobicity, low bioavailability, and rapid metabolism of Que (Lawson, 2023), suitable drug delivery systems is urgently needed to increase its bioavailability.

Liposomes are renowned for their unique structures and functions in various drug delivery systems (Large et al., 2021; Cheng et al., 2021; Yan et al., 2023). It has become a prominent choice among drug carriers due to its biocompatible composition, excellent biocompatibility, biodegradability, which subsequently enhance the drug stability (Shah et al., 2020; Antimisiaris et al., 2021). Que within liposomes may enhance the stability and solubility of Que, overcome challenges of hydrophobicity, limited bioavailability, and rapid metabolism. Additionally, liposomes can be directly delivered into the lungs via intratracheal (i.t.) administration avoiding the lung clearance and enzymatic breakdown and minimizing adverse effects on other parts of the body on account of its biodegradability (Wang et al., 2021; Shen and Minko, 2020; Tong et al., 2024). Therefore, liposomes combined with Que may be a good approach for COPD treatment.

In this study, a novel Que liposome (Que-lipo) was prepared and administrated via i.t. with CS induced COPD mice. The inflammation, pulmonary function and pathological condition of lung were improved after i.t. administration of Que-lipo. The underlying therapeutic mechanisms against lung damage of Que-lipo mediated by inflammasome activation and reverse fibrosis related to pathological and biochemical processes, which will provide new understanding of nanotechnology to enhance Que’s therapeutic effect, and novel perspectives for advancing therapeutic approaches for COPD.

## 2 Materials and methods

### 2.1 Materials

Que was purchased from Merck. Lecithin and cholesterol were purchased from Aladdin. DSPE-PEG2000 was purchased from Biomatrik. A549 cells and Beas-2B cells were derived from National Collection of Authenticated Cell Cultures. Enzyme-Linked Immunosorbent Assay (ELISA) detection kits were used from Shanghai Enzyme-linked Biotechnology Co., Ltd. The fetal bovine serum and DMEM for cell culture were purchased from Gibco. The antibodies of Bcl-2, caspase-3 and caspase-7 were purchased from Proteintech Group, Inc. Antibodies against TGF-β, ROCK, Rho and α-SMA were purchased from Abcam.

### 2.2 Preparation of Que-lipo

Que-lipo were prepared based on thin film dispersion method. Briefly, lecithin/cholesterol/Que/DSPE-PEG2000 were dissolved in chloroform-methanol (1:1, v/v) mixture at a mass ratio of 13:4:1:6. The phospholipid film was formed by vacuum distillation at room temperature for 2–3 h in a rotary evaporator. Then, 50–150 mL of 5% glucose solution was added to the round-bottom flask containing the phospholipid film for ice bath ultrasound until the liposomes on the flask wall were completely detached and uniformly dispersed in the glucose solution. After that, the liposomes particles were repeatedly frozen and thawed with liquid nitrogen for more than 20 times. Finally, the unencapsulated Que large particles and unstable liposomes were removed by centrifugation at 6,000 rpm at 4°C for 10 min.

### 2.3 Characterization and physicochemical properties of Que-lipo

#### 2.3.1 Transmission electron microscopy (TEM)

The morphology and size of Que-lipo were observed by TEM. Before observation, a small amount of Que-lipo aqueous solution was diluted. Then it was added dropwise to the special copper mesh covered with carbon film, and then negatively stained with 2% phosphotungstic acid solution, and observed after drying. The image was shot at ×50,000 magnification.

#### 2.3.2 Particle size and polydispersity index (PDI)

The Que-lipo aqueous solution was diluted two times with ultrapure water. The particle size and PDI of Que-lipo were measured by laser particle size analyzer. The particle size measurement range is 2–3,000 nm. The measurement angle is 90°, and the test temperature is 25°C. After measured, the Que-lipo was stored in a refrigerator at 4°C.

#### 2.3.3 Determination of Que concentration and encapsulation efficiency (EE)

The prepared Que-lipo was mixed with 90% ethanol solution for 10 min to destroy the membrane structure of nanoliposomes and release the encapsulated Que. The concentration of Que in the solution was quantified using ultraviolet spectrophotometry. The standard curve of Que was calibrated by ultraviolet spectrophotometry. The EE of Que was determined by indirect method (Hao et al., 2017). The unencapsulated Que and the Que-lipo were separated by high-speed centrifugation. The absorbance of the solution was measured at 371 nm after dilution. The encapsulation efficiency was calculated via Equation 1. A0 is the total amount of Que, and A1 is the content of unencapsulated Que in the precipitate.

$$\left[EE\;\left(\%\right)=\left(A0-A1\right)/A0\times 100\%\right]$$ (1)

#### 2.3.4 *In vitro* release of Que

Que-lipo were placed in a dialysis bag with a diameter of 22 mm and a length of about 10 cm. The air in the bag was removed. And the two ends were tied tightly with a wire rope, coiled and fixed on the dissolution instrument stirring paddle. The stirring paddle was placed at about 1.5 cm from the bottom of the dissolution cup. 250 mL of PBS was used as the release medium. The temperature was maintained at (37.0 ± 0.5)°C, and the stirring paddle was rotated to 50 r/min. 2 mL sample was removed at 1, 2, 4, 6, 8, 12, 24, 36 and 48 h, respectively. The same volume of release medium was added at the same time. The cumulative release percentage of Que at each time was calculated by indirect method before filtering with 0.45 μm microporous membrane (Hao et al., 2017).

### 2.4 Cell viability and cellular uptakes

#### 2.4.1 Cell viability

A549 and Beas-2B cells were completely cultured with DMEM containing 10% FBS. The cells were cultured at 37°C, 5% carbon dioxide, digested with 0.125% trypsin (containing 0.01% EDTA) and passaged once every 2–3 days. The biocompatibility was detected by cell counting kit-8 (CCK-8) kit. The specific steps are as follows: A549 or BEAS-2B cells in logarithmic growth phase were prepared into single cell fluid, and the density of 1 × 10^4^ cells per well was uniformly planted in 96-well plates. After incubation in a carbon dioxide incubator for 12 h, the growth medium was replaced with 100 uL of fresh medium containing different concentrations of Que-lipo (2.5, 1.25 and 0.625 μM) or Que (2.5, 1.25 and 0.625 μM). After the end of the exposure, 10% volume of CCK-8 was added to each well and incubated at 37°C for 2–3 h. The optical density was measured at 450 nm wavelength using a microplate reader.

#### 2.4.2 Cellular uptakes

The cellular uptake behaviors of Que-lipo were studied in A549 cells. A549 cells (1 × 10^5^ per well) were seeded in a six well plate, incubated at 37°C for 24 h, and then treated with the DiI labeled Que-lipo at a dose of 5 μg/mL Que equivalent, respectively. After 2 h coincubation, the cells were imaged by confocal laser scanning microscope (CLSM).

### 2.5 Animal experiment and construction of COPD model mice

The C57BL/6 male mice (8-week-old) were provided by Zhejiang Vital River Laboratory Animal Technology Co., Ltd. (China), and the experimental animal production license number was SCXK (Zhe) 2021-0006. The feeding environment temperature was controlled at (22 ± 2)°C, and the experiment was carried out after 7 days of adaptive feeding. The animal experiment operation involved in this experiment was approved by the Laboratory Animal Management and Ethics Committee of China Tobacco Quality Supervision and Test Center (CTQTC-SYXK-2023004).

Thirty mice were randomly divided into control group and smoking model group. After adaptive feeding for 1 week, the smoke exposure group was exposure with smoke to induce COPD model, while the control group was fed in normal environment for 24 weeks. The mice in the smoking group were passive smoked twice in the box for 60 min each day (the mice in the first week of smoking were given gradually increased adaptation time to the mice), followed by four cigarettes (3R4F) each time, burning in two batches, igniting and putting into the smoking box, self-burning, two times of smoking interval ≥ 5 h.

### 2.6 Dosage and method of administration in animal experiments

Two administration methods including i.p. and i.t. were used to explore the difference in the efficacy of Que’s efficacy against COPD. The COPD mice were divided into four groups including model group, Que (i.p.) group (50 mg/kg), Que-lipo (i.p.) group (0.3 mg/kg) and Que-lipo (i.t.) group (0.3 mg/kg). The Que-lipo (i.t.) group were administrated as follows. Firstly, the mice were anesthetized by intraperitoneal injection of pentobarbital sodium (40 mg/kg). Then the mice were prone to the tracheal intubation platform, and the light source was aimed at the neck. The tongue was gently pulled out with a flat-headed tweezer, and a small amount of cotton ball was taken with another tip to clean its laryngopharyngeal saliva. The light source was adjusted to be clearly visible at the tracheal orifice, showing a bright dot. The drug delivery device is a lung quantitative atomizer (Beijing Huironghe Technology Co., Ltd.). The needle of the pre-prepared drug delivery device is inserted into the tracheal orifice under visual conditions, and the liposomes liquid was rapidly pushed and ejected at high speed to be deposited.

### 2.7 Plethysmography

An unrestrained whole-body plethysmograph (Buxco) was used to evaluate the pulmonary function of the mice. The main pulmonary function index was enhanced pause (Penh). Besides expiration time (Te) and breathing frequency (F) were also measured. Before each test, the mouse instrument was adapted for 90 min and then tested for 20 min. During the test, clean air and free activity space were guaranteed for the weight of the mice.

### 2.8 Enzyme-linked immunosorbent assay (ELISA) experiments

The lung tissue homogenate was homogenized and centrifuged to obtain the supernatant. The contents of TGF-β1, α-SMA, superoxide dismutase (SOD), Glutathione peroxidase (GSH-Px) and MPO were detected in strict accordance with the instructions of ELISA kits.

### 2.9 Immunohistochemistry

The wax blocks used for histopathology were prepared into the required slides, and then the tissue antigens were recovered by enzymatic digestion. Based on the principle of antigen-specific binding, a labeling chromogenic agent was developed by chemical reaction to determine the antigens in tissues and cells, and their localization, qualitative and quantitative studies were carried out.

### 2.10 Histopathology and immunohistochemistry

After the last measurement of lung function, the mice were sacrificed by cervical dislocation. The lung tissue was immersed in a 4% paraformaldehyde phosphate buffer for over 48 h, subsequently dehydrated, embedded in paraffin, and sectioned into 4 μm paraffin sections. The slices were performed with H&E dye and Masson’s Trichrome Stain to observe the morphological changes and the induction of collagen fibers. In addition, the slices were restored by antigen retrieval and stained with specifically antigens. DAB was subsequently used to obtain visible precipitate at the site of the antigen. At last, all the slices were observed with a light microscope.

### 2.11 Reverse Transcription - Polymerase Chain Reaction (RT-PCR)

About 100 mg of mice lung tissue was cut into pieces and added into 1 mL of TRIzol solution containing RNA degrading enzyme. After grinding and lysis, chloroform was added to extract total RNA from lung tissue. The concentration and purity of RNA were determined by ultraviolet spectrophotometry after purification. Reverse transcription reaction was performed using qPCR kit. kit. The amplification conditions were: 50°C 2 min, 95°C 2 min, 95°C 15 s, 60°C 32 s, 40 cycles. After amplification, the expression of the target genes and internal reference GAPDH was calculated by 2^−ΔΔCT^ relative quantification.

### 2.12 Biodistribution

After the mice were administrated with Que or Que-lipo, the target organs (about 0.2 g) were removed and homogenized with ice-cold physiological saline (5 mL/g tissue). Subsequently, 50 μL of the tissue homogenate was transferred to a 1.5 mL EP tube, followed by the addition of 20 μL of 2 mg/mL vitamin C solution and 25 μL of β-glucuronidase/arylsulfatase. The mixture is thoroughly mixed and enzymatically digested in a 37°C water bath for 30 min. This was followed by the addition of 25 μL of Que-D5 internal standard solution (0.8 μg/mL) and 20 μL of 2.5 mol/L hydrochloric acid solution, which was then vortexed for 2 min. Subsequently, 1,000 μL of ethyl was added to the solution, followed by vortexing for 5 min and centrifugation at 11,000 rpm at 4°C for 10 min. Finally, 500 μL of the supernatant should be extracted, evaporated to dryness under vacuum, and reconstituted with 100 μL of mobile phase. The resulting supernatant was utilized for liquid chromatography-mass spectrometry (LC-MS) analysis.

The mass spectrometry conditions were as follows: the ionization voltage −4500 V, temperature 500°C, the curtain gas pressure 45.0 psi, the collision gas pressure 8 psi, the nebulizer gas pressure 50 psi, and an auxiliary heating gas pressure 55 psi, and an auxiliary heating gas pressure of 55 psi. Chromatographic separation was conducted using an Agilent Poroshell 120 EC-C18 column (3.0 × 100 mm, 2.7 μm) with a mobile phase consisting of 0.1% formic acid in a mixture of water and acetonitrile (70:30, v/v). The column temperature was maintained at 30°C, the flow rate was set at 0.3 mL/min, and the injection volume was 5 μL.

### 2.13 Statistical analysis

The data were expressed as mean ± standard deviation of three independent experiments. All statistical analyses were performed using graphpad software. One-way analysis of variance (ANOVA) and Tukey’s multiple comparisons were used to assess the differences between the samples. All significant differences were based on a 0.05 probability level.

## 3 Results

### 3.1 Characterization of Que-lipo

**FIGURE 1:**
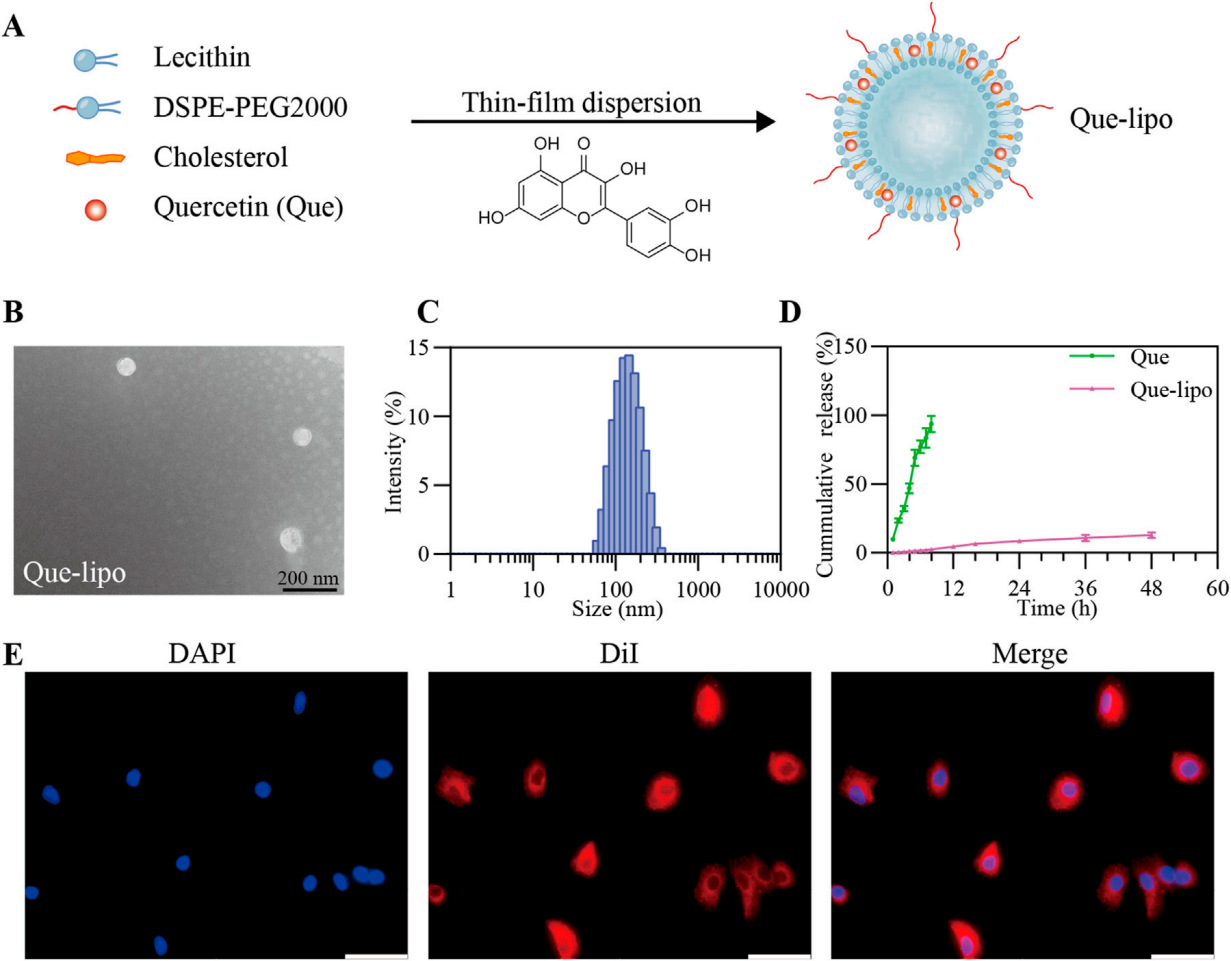
Fabrication and characterization of Que-lipo. **(A)** Schematic illustration of Que-lipo preparation. **(B)** TEM pictures of Que-lipo. **(C)** DLS dimensional distribution of Que-lipo. **(D)** Cumulative *in vitro* release of Que and Que-lipo at pH 7.4. Data represents mean ± SD (n = 3). **(E)** Cellular uptake behaviors of Que-lipo in A549 cells. Scale bar = 50 μm.

Que-lipo was prepared using the film dispersion method, as illustrated in Figure 1A. DSPE-PEG2000 was used to improve the stability of liposomes and the encapsulation efficiency of Que, thereby enhancing the drug delivery efficacy. We quantified the drug loading and EE of Que-lipo as (5.47 ± 0.15) % and (91.26 ± 3.18) %, respectively. Using TEM and DLS, we confirmed that Que-lipo exhibited a well-defined spherical structure and a uniform particle size distribution. The average size of Que-lipo observed in TEM was about 74 nm (Figure 1B), while the hydrated particle size was 128 nm (Figure 1C). And the results indicated that the PDI of Que-lipo was below 0.25.

### 3.2 *In vitro* drug release

The release of Que-lipo *in vitro* was evaluated by dialysis membrane bag diffusion method. As depicted in Figure 1D, the cumulative release of Que-lipo reached 3.1% within the initial 0–8 h, 9.2% at 24 h, and 13.6% at 48 h, indicating a gradual and consistent release pattern compared to free Que. This sustained-release property *in vitro* promoted Que maintain an optimal concentration, potentially enhancing the therapeutic efficacy and reducing the risk of toxicity.

### 3.3 Cytotoxicity assay and *in vitro* cellular uptakes of Que-lipo

The potential toxicity of Que and Que-lipo in A549 and Beas-2B cell lines was evaluated by CCK-8 assay, aiming to determine their biocompatibility for pulmonary application. As shown in Supplementary Figure 1, Que and Que-lipo exhibited minimal cytotoxicity on these cell lines, with cell viability exceeding 90% at Que concentrations up to 2.5 μM. Additionally, we investigated the internalization of Que-lipo *in vitro* using CLSM. Que-lipo were labeled with DiI fluorescent dye, which effectively bound to the liposome’s lipid bilayer and emitted a bright red fluorescence. After a 2-hour co-incubation with A549 cells, the CLSM images in Figure 1E showed that the red fluorescence signal predominantly localized within the cytoplasm.

### 3.4 The construction of COPD mouse model

**FIGURE 2:**
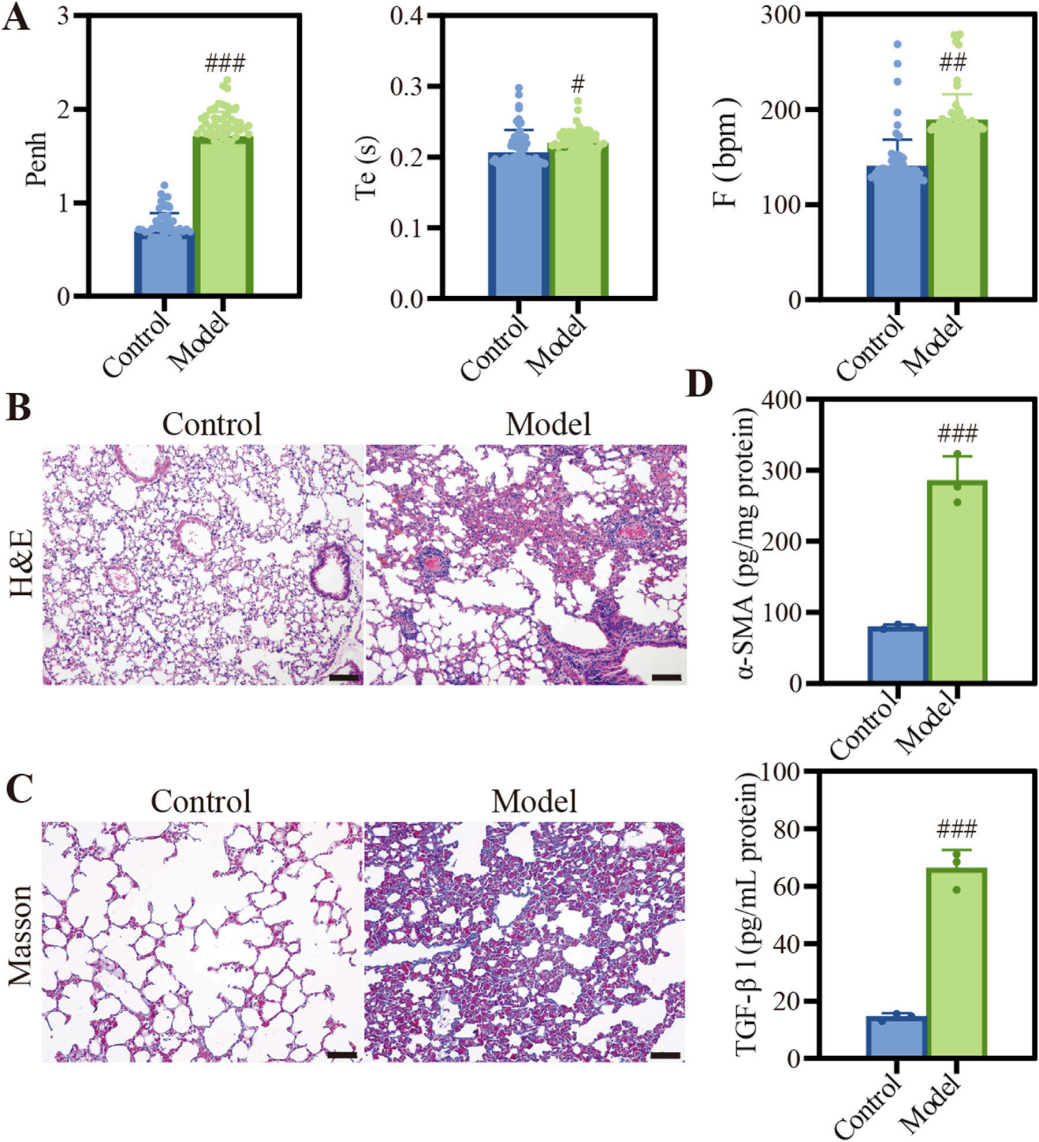
Characterization of COPD model induced by CSE. **(A)** Description of Penh, Te, and F. Data were analyzed by One-way ANOVA. ^#^ *p* < 0.05, ^##^ *p* < 0.01, ^###^ *p* < 0.001 versus control; **(B)** H&E staining images of different groups, Scale bar: 100 μm. **(C)** Masson staining images of different groups, Scale bar: 50 μm. **(D)** Quantification of α-SMA and TGF-β1 protein expression. Data were analyzed by One-way ANOVA. ^#^ *p* < 0.05, ^##^ *p* < 0.01, ^###^ *p* < 0.001 versus control. Data represents mean ± SD (n = 3).

We established a COPD mouse model by exposing the animals to CS, which was a well-established method to mimic the key features of the disease. As shown in Figure 2A, the model group exhibited significantly elevated Penh, Te and F compared to the control group (Figure 2A). These results aligned with the typical features of diminished lung function observed in individuals with COPD (Ding et al., 2024). Histopathological examination revealed significant heterogeneity in alveolar size within the model group, with many instances of alveolar rupture and fusion (Figure 2B). Additionally, we observed thickening and proliferation of fibrous tissue in the bronchial walls, along with luminal narrowing and significant infiltration of inflammatory cells. Masson staining of the lungs in COPD mice showed a deepened blue color, indicating the presence of pulmonary fibrosis and structural alterations (Figure 2C). This finding was supported by the increased expression levels of pulmonary fibrosis markers Alpha-smooth muscle actin (α-SMA) and TGF-β1 (Figure 2D).

### 3.5 Que-lipo repaired lung function in COPD mice

**FIGURE 3:**
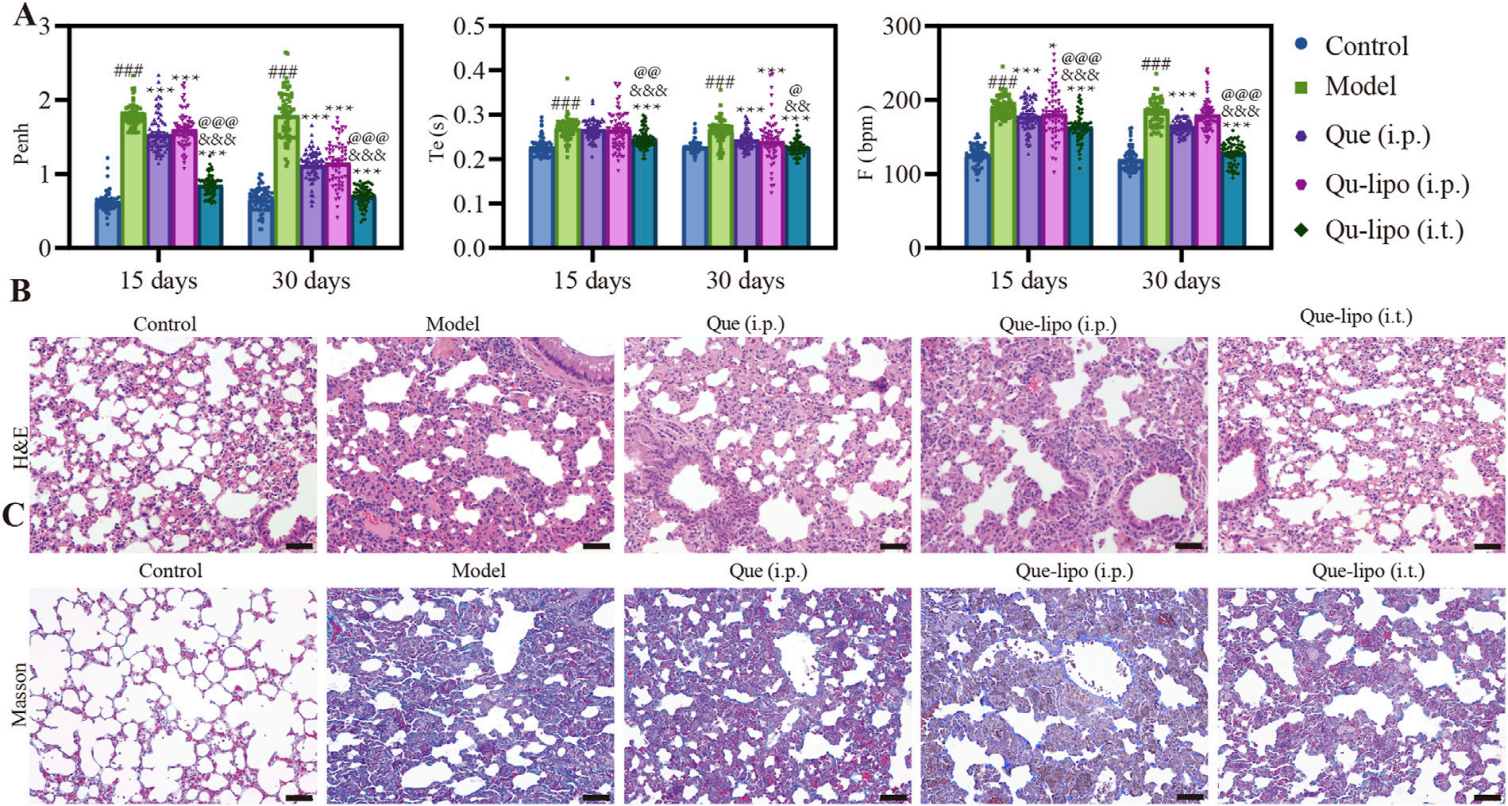
Effects of different dosing modalities in the treatment of COPD. **(A)** Description of Penh, Te, and F, Data were analyzed by One-way ANOVA. ^#^ *p* < 0.05, ^##^ *p* < 0.01, ^###^ *p* < 0.001 versus control; *p < 0.05, **p < 0.01 versus model; ^&^ *p* < 0.05 versus Que (i.p.); ^@^ *p* < 0.05, ^@@^ *p* < 0.01, ^@@@^ *p* < 0.001 versus Que-lipo (i.p.); Data represents mean ± SD (n = 69). **(B)** H&E staining images of different groups, Scale bar: 50 μm. **(C)** Masson staining images of different groups, Scale bar: 50 μm.

To further assess the therapeutic effectiveness of Que-lipo for COPD, we investigated the impact of two distinct administration routes on therapeutic outcomes in COPD mice, namely, i.t. and i.p. In addition, the treatment with a high dose of free Que via i.p. was also compared. After 30-day of treatment, the results in Figure 3A showed Que-lipo (i.t.) had significant effect on the parameters Penh, Te, and F. The therapeutic efficacy of Que-lipo (i.t.) was notably superior to Que-lipo (i.p.) and Que (i.p.) after 15 days of treatment. Lung function gradually approaching normal levels with extended therapy. Besides, Que-lipo (i.t.) enhanced alveolar uniformity and strengthens the integrity of alveolar wall structures, while also reducing the infiltration of inflammatory cells (Figure 3B). Furthermore, masson staining indicated that i.t. administration of Que-lipo more effectively achieved a significant decrease in the fibrotic area (Figure 3C). The tissue lesion score of histopathological and masson were scored in Table 1 according to the literature (Szapiel et al., 1979; Ashcroft et al., 1988). These findings collectively supported i.t. as a superior approach for enhancing lung function in mice with COPD.

**TABLE 1:** Histopathological and masson score in mice in different groups (mean ± SD, n = 3).

	Control	Model	Que (i.p.)	Que-lipo (i.p.)	Que-lipo (i.t.)
Histopathological Score ( $\bar{X}$ ± SD)	1.3 ± 1.0	3.9 ± 1.1	3.4 ± 1.2	4.3 ± 1.7	2.8 ± 0.9
Masson Sore ( $\bar{X}$ ± SD)	0 ± 0	1.7 ± 0.6	1.3 ± 0.6	1.0 ± 0.8	0.8 ± 0.3

### 3.6 Liposomes improved the bioavailability of Que in COPD mice

To deepen understanding of the observed therapeutic effects, we utilized LC-MS to precisely quantify the distribution of Que content following i.t. of Que-lipo and i.p. of Que at 24 h post-treatment. As illustrated in Supplementary Figure 2, the concentration of Que in the lungs after Que-lipo (i.t.) was 19.0 ID%/g tissue, significantly higher than the 5.5 ID%/g tissue with Que (i.p.) and 3.7 ID%/g tissue with Que-lipo (i.p.). These results confirmed the advantages of liposomes for enhancing the bioavailability of Que. Moreover, the substantial difference highlighted the efficacy of i.t. in elevating pulmonary drug concentration, potentially offering a more effective intervention in COPD progression. Additionally, i.t. of Que-lipo circumvented the first-pass effect, ensuring more efficient delivery of Que directly to the lungs. These findings provided compelling evidence for the i.t. as an effective therapeutic approach for COPD and underscored the critical role of the drug administration route in optimizing treatment outcomes.

### 3.7 The intratracheal delivery of Que-lipo improved the oxidative stress and inflammation symptoms in COPD mice

**FIGURE 4:**
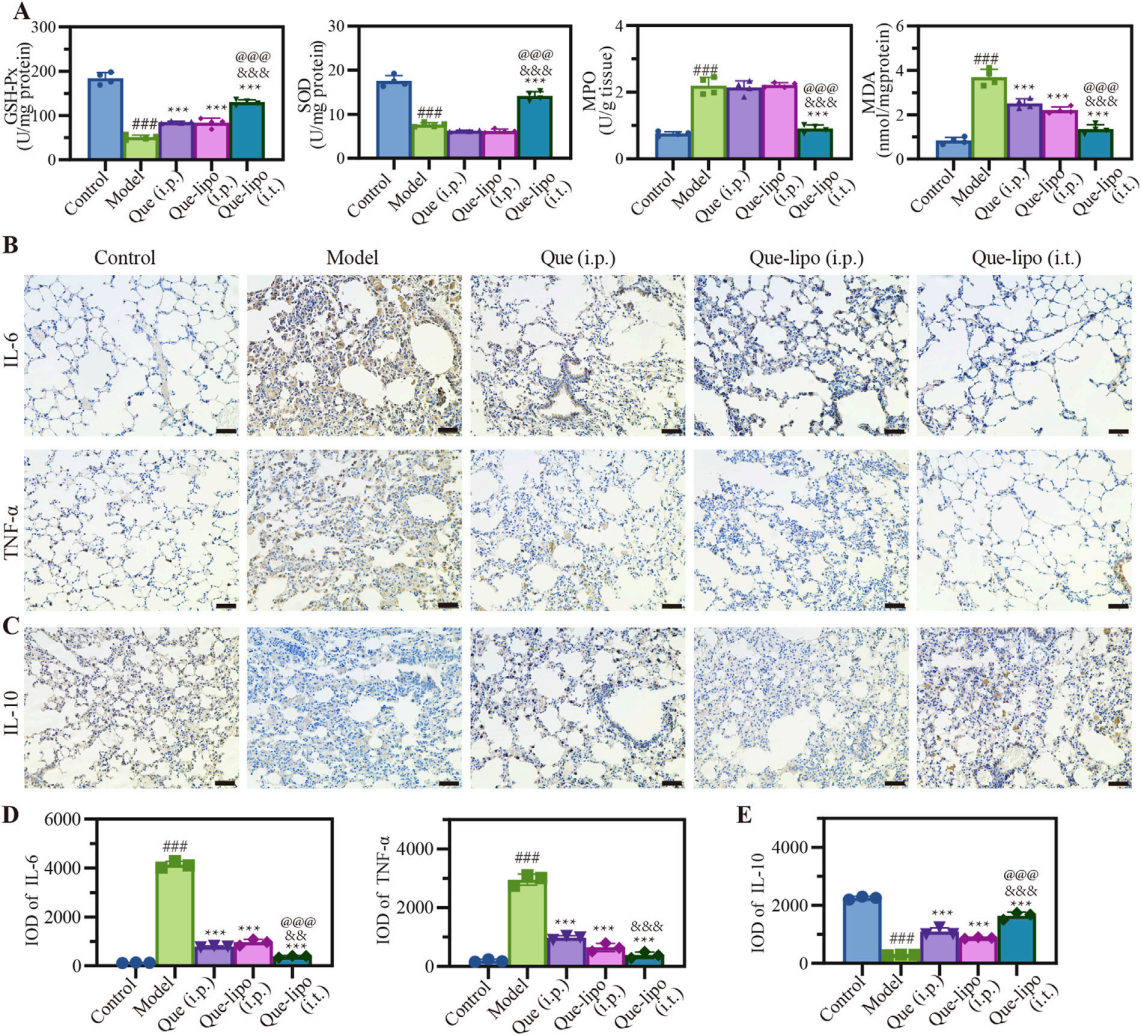
Effects of Que and Que-lipo on oxidative stress and inflammatory markers in the treatment of COPD. **(A)** Levels of GSH-Px, SOD, MDA, and MPO in the lungs. **(B)** Immunohistochemical analysis of **(B)** IL-6, TNF-α and **(C)** IL-10 in the lungs. Mean IOD values of **(D)** IL-6, TNF-α and **(E)** IL-10 in the lungs in lungs. Data were analyzed by One-way ANOVA. ^###^ *p* < 0.001 versus control; ***p < 0.001 versus model; ^&&&^ *p* < 0.001 versus Que (i.p.); ^@@@^ *p* < 0.001 versus Que-lipo (i.p.); Data represents mean ± SD (n = 4).

Systemic smoke exposure induces pronounced inflammatory responses in both pulmonary tissue and the overall physiology in COPD mice. GSH-Px and SOD are crucial antioxidant enzymes that actively combat oxidative stress by neutralizing free radicals and protecting cells from oxidative damage. As shown in Figure 4A, the model group exhibited significant differences in various detection indicators when compared with the control group, reflecting the typical biochemical alterations in COPD. The oxidative activity of GSH-Px and SOD decreased in COPD mice due to heightened oxidative stress. Given their frequent co-occurrence, MDA and MPO levels were significantly elevated. Following treatment, the Que-lipo (i.t.) showed a statistically significant therapeutic effect compared to the model group (*p* < 0.001). In contrast, Que (i.p.) did not exhibit significant differences across all detection indicators.

Additionally, post-treatment assessment of inflammatory markers in the lungs provided additional insights into the inflammatory status of COPD mice. We next assessed the changing expression levels of pro-inflammatory factors including IL-6 and TNF-α, and anti-inflammatory factors IL-10 in lungs by immunohistochemistry. The immunostaining results presented in Figure 4B indicated a significant elevation in IL-6 and TNF-α levels within the model group, which were markedly reduced following treatment with Que-lipo. These levels were significantly higher compared to the control group (*p* < 0.001). The quantitative analysis of IL-6 and TNF-α in Figure 4D showed that significant differences were found in Que-related groups (*p* < 0.001). In contrast, IL-10 exhibited an opposite trend in Figures 4C,E. Upon treatment, the Que-lipo formulation, when administered intratracheally (i.t.), showed a significant therapeutic impact, starkly divergent from the model group (*p* < 0.001). This effect was even more pronounced when compared to the i.p. administration of Que and Que-lipo.

### 3.8 The intratracheal delivery of Que-lipo regulated the expression levels of apoptosis proteins in COPD mice

**FIGURE 5:**
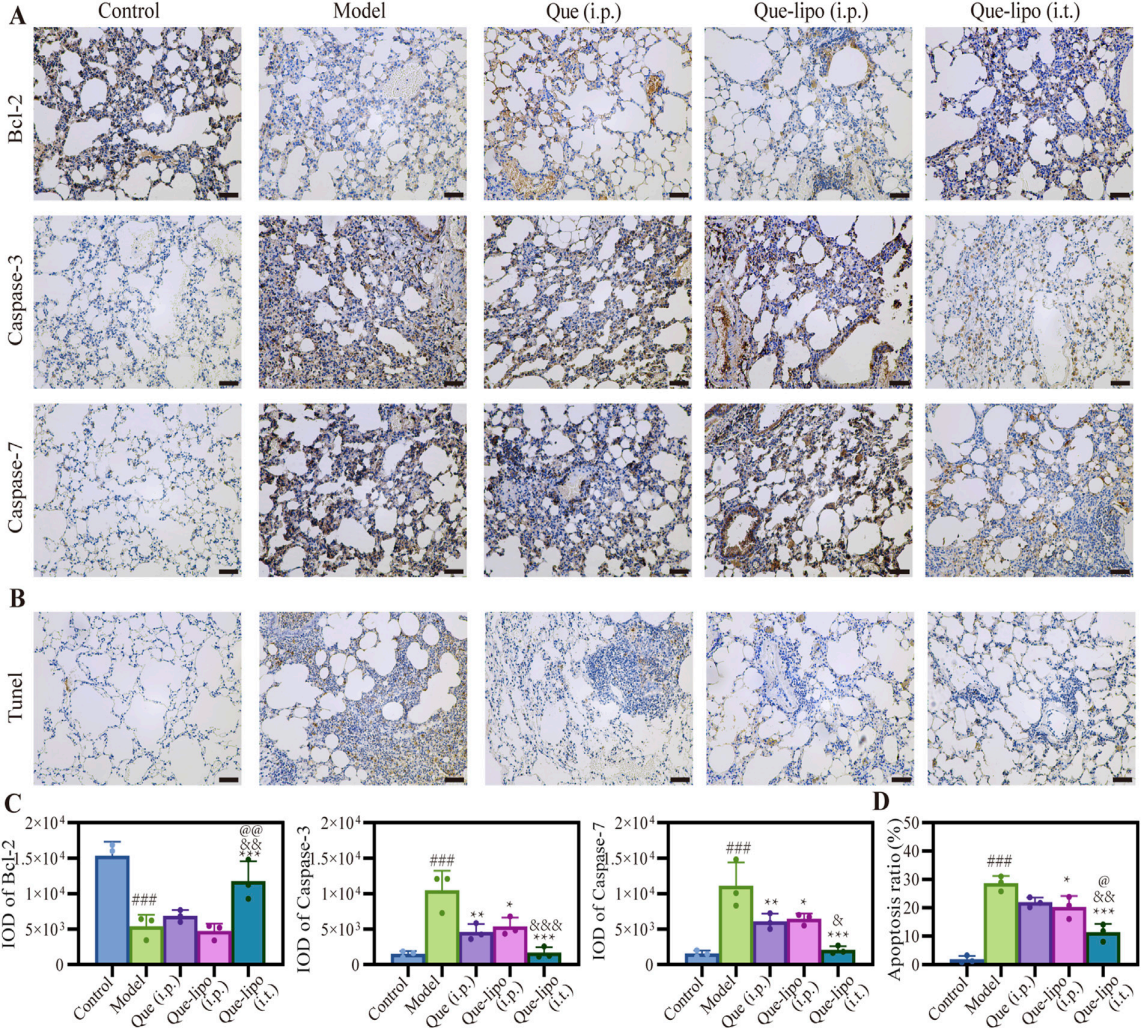
Apoptosis inhibition of Que and Que-lipo in the lung of COPD mice. **(A)** Immunohistochemical analysis and **(B)** mean IOD values of Bcl-2 and caspase-3/7 in lungs. Scale bar: 50 μm; **(C)** TUNEL staining and **(D)** apoptosis ratio in lungs. Scale bar: 50 μm; Data were analyzed by One-way ANOVA. ^###^ *p* < 0.001 versus control; *p < 0.05, **p < 0.01, ***p < 0.001 versus model; ^&^ *p* < 0.05, ^&&^ *p* < 0.01, ^&&&^ *p* < 0.001 versus Que (i.p.); ^@^ *p* < 0.05, ^@@^ *p* < 0.01 versus Que-lipo (i.p.); Data represents mean ± SD (n = 3).

As a chronic lung disease, COPD is closely associated with apoptotic responses (Lu et al., 2021). Thus, we assessed the expression patterns of apoptotic proteins in COPD mice following various treatment. Bcl-2 and caspase-3/7 levels were evaluated in COPD using immunohistochemical staining. The results in Figures 5A,C illustrated that levels of the anti-apoptotic protein Bcl-2 were considerably reduced in the COPD group compared to the control group (*p* < 0.001). Conversely, the levels of the pro-apoptotic protein caspase 3/7 were significantly elevated (*p* < 0.001). And the results found that the number of positive cells (brown-yellow) in COPD mice was significantly higher compared to normal mice (Figure 5B), indicating heightened apoptotic activity in COPD. Both i.t./i.p. of Que-lipo and i.p. of Que exhibited therapeutic benefits for COPD post-treatment. It was demonstrated with a notable significant intensity and IOD difference when compared to the model group. Additionally, the results in Figures 5C,D suggested that i.t. of Que-lipo yielded superior therapeutic effects over the treatment of Que (i.p.) and Que-lipo (i.p.). The TUNEL assay results, as depicted in Figures 5B,D, corroborated the protein expression findings (Figures 5A,C). It indicated that both treatment modalities led to a significant reduction in cellular apoptosis. Notably, Que-lipo with i.t. exhibited a more pronounced effect.

### 3.9 The impact of intratracheal Que-lipo administration on inflammasomes activation in COPD mice

**FIGURE 6:**
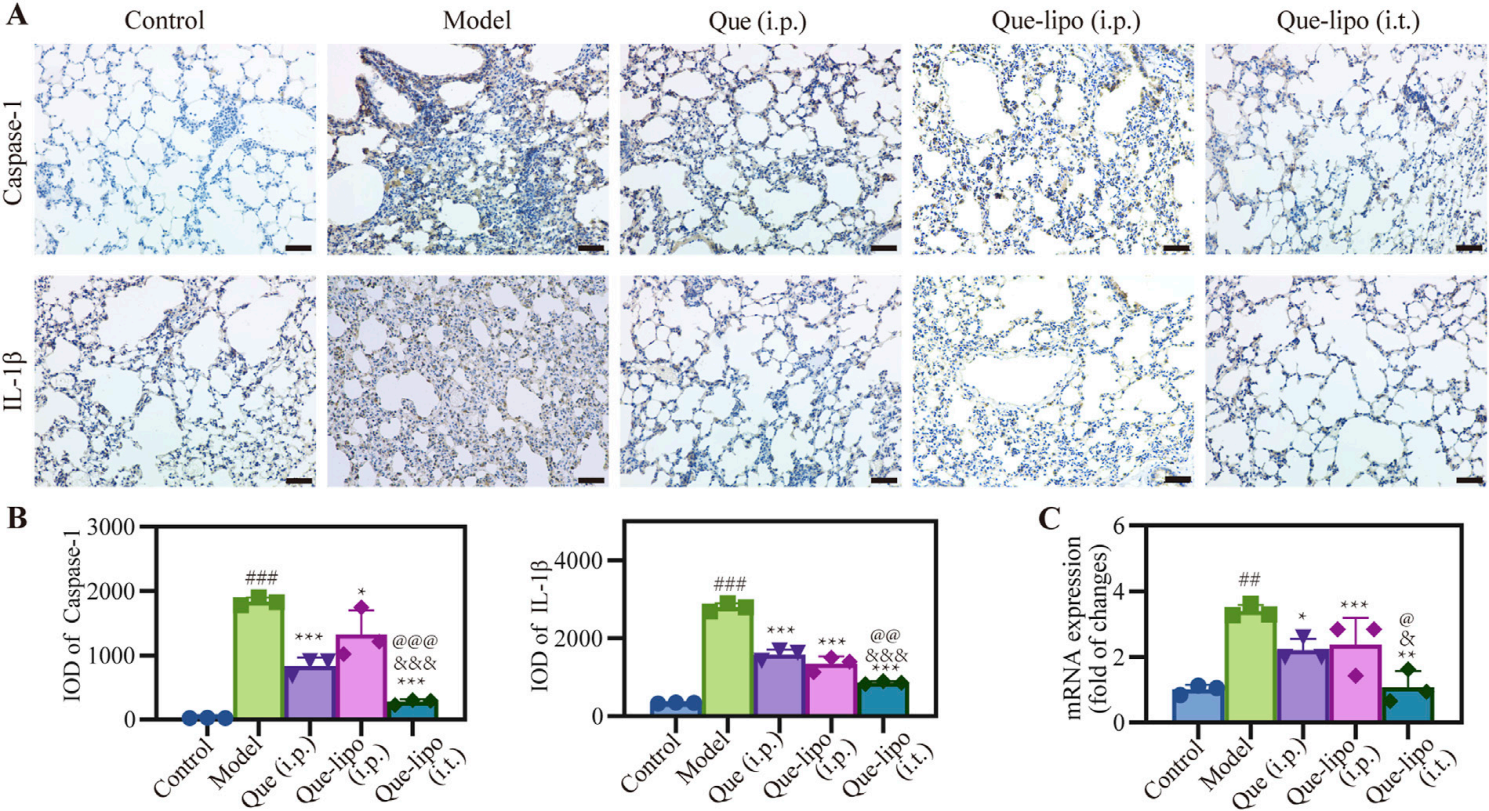
Effects of Que and Que-lipo on the expression of key inflammatory factors in the lung of COPD mice. **(A)** Immunohistochemical analysis and **(B)** mean IOD of caspase-1 and IL-1β in lungs. Scale bar: 50 μm; **(C)** mRNA expression of NLRP3; Data were analyzed by One-way ANOVA. ^##^ *p* < 0.01, ^###^ *p* < 0.001 versus control; *p < 0.05, **p < 0.05, ***p < 0.001 versus model; ^&^ *p* < 0.05, ^&&&^ *p* < 0.001 versus Que (i.p.); ^@^ *p* < 0.05, ^@@^ *p* < 0.01, ^@@@^ *p* < 0.001 versus Que-lipo (i.p.); Data represents mean ± SD (n = 3).

The escalation of inflammatory responses is often marked by an increased expression of critical factors within the inflammatory pathway. After treatment with Que and Que-lipo through different administration methods, the pulmonary expression levels of significant inflammatory biomarkers, specifically caspase-1 and IL-1β, were assessed using immunohistochemical techniques. The findings presented in Figure 6A demonstrated a significant upregulation of these factors in the COPD model group relative to the control group. This result underscored the active nature of inflammatory response in COPD. Expanding upon these insights, Figure 6B presented a detailed quantitative integrated optical density (IOD) analysis for caspase-1 and IL-1β. PCR was then employed to analyze the expression of inflammatory-related markers of NLRP3. The results in Figure 6C aligned with the trends observed for caspase-1 and IL-1β. This comprehensive analysis further confirmed the elevated inflammatory activity within the model group. Notably, all treated groups reduced level of inflammatory factors, of which Que (i.t.) had the optimal effect versus Que (i.p.) or Que-lipo (i.p.). The comparative analysis of the therapeutic efficacy revealed that Que-lipo (i.t.) displayed more pronounced benefits, particularly in terms of direct pulmonary drug intervention.

### 3.10 The improvement of intratracheal Que-lipo on fibrosis molecular level in COPD mice

**FIGURE 7:**
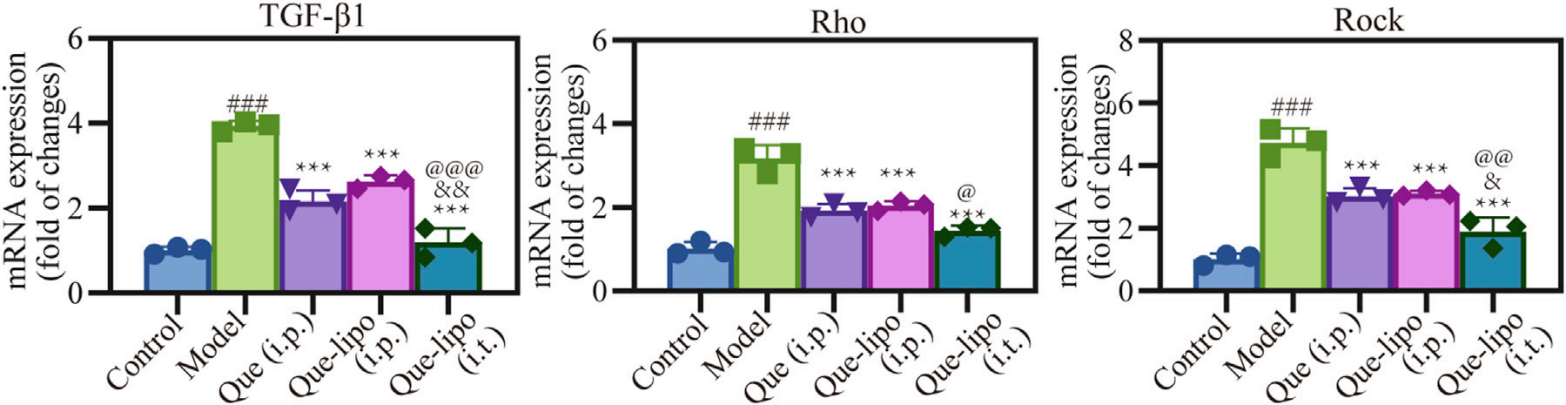
Effects of Que and Que-lipo on the expression of fibrosis signaling molecules in the lung of COPD mice. mRNA expression of TGF-β1, Rho, and Rock; Data were analyzed by One-way ANOVA. ^###^ *p* < 0.001 versus control; **p < 0.01, ***p < 0.001 versus model; ^&^ *p* < 0.05, ^&&^ *p* < 0.01 versus Que (i.p.); ^@^ *p* < 0.05, ^@@^ *p* < 0.01, ^@@@^ *p* < 0.001 versus Que-lipo (i.p.); Data represents mean ± SD (n = 3).

The RT-PCR was used to investigate the molecular mechanisms underlying the therapeutic potential of Que-lipo in the treatment of COPD. Figure 7 presented the mRNA expression of key factors involved in the pulmonary fibrosis pathway. It was observed that the high mRNA expression of TGF-β1, Rho and ROCK in the model group compared with the control group (*p* < 0.001), suggesting that pulmonary fibrosis was indeed a predominant feature of COPD. After Que (i.p.), Que-lipo (i.p.) and Que-lipo (i.t.) treatment, the mRNA expression of TGF-β1, Rho and ROCK decreased significantly (*p* < 0.001). The result indicated both Que and Que-lipo had potential as an effective therapy for COPD. Moreover, the comparative analysis between Que-lipo (i.p.) and Que-lipo (i.t.) showed that the more downregulated mRNA level of Que-lipo via i.t. was observed if than Que-lipo via i.p. (*p* < 0.05). This strengthens the notion that the direct delivery to the lungs provide a more effective therapeutic options than systemic injection in pulmonary diseases.

## 4 Discussion

Due to the inhalation of noxious particles or gases, lung impairment in CS induced COPD is characterized by an exacerbated inflammatory response and a disrupted redox balance within the lung tissue. Current pharmaceutical interventions for COPD primarily comprised by glucocorticoids and bronchodilators, which provide symptomatic relief but are insufficient in significantly altering the disease’s progression. Therefore, natural products with antioxidant and anti-inflammatory properties have aroused great research interest. In this study, we demonstrated that Que-lipo effectively ameliorates oxidative stress and changes in lung morphology and function induced by CS exposure.

First, we showed the preparation and detailed characterization of Que-lipo in Figure 1. Que-lipo were prepared based on the thin film hydrated dispersion method (Figure 1A). The method of thin film dispersion was selected due to advantages of simplicity, high efficiency and material saving. The prepared Que-lipo had high embedding efficiency and drug loading capacity. It demonstrated that Que was successfully loaded into liposomes, which greatly improved the drug delivery efficiency. The EE and drug loading were 91.26% ± 3.18% and 5.47% ± 0.15%, respectively. The nanoscale dimensions of liposomes are crucial for their application in biomedicine. As depicted in Figures 1B,C, the Que-lipo morphology and size were further studied by TEM and DLS. Que-lipo exhibited a relatively uniform spherical morphology, as measured by TEM. The average size of Que-lipo measured from the TEM images was 75 nm (Figure 1B). DLS studies showed that the hydrodynamic diameter of Que-lipo was 128 nm with a PDI of 0.25. The Que-lipo sizes measured by TEM agreed well with that measured by DLS. Of note, the particles sizes in TEM were smaller than in DLS, which may be due to the different measurement principles in different techniques. The liposome particle sizes were obtained in solution with DLS and in a dry state in TEM.

Que-lipo exhibited a smooth release rate and longer release time compared to unmodified Que *in vitro*. The results in Figure 1D exhibited a sustained-release pattern during the 48-hour release study with about 13.6% cumulative drug release, whereas Que showed nearly 100% release after 12 h. It was concluded that the liposomal encapsulation of Que significantly enhanced the controlled release of the drug, which was a desirable property for drug delivery systems. The extend release profile of Que-lipo can provide a more prolonged therapeutic effect in COPD, reducing the frequency of administration and potentially improving compliance. The *in vitro* cytotoxicity assessment in A549 and Beas-2B cell lines suggested that Que-lipo had negligible cytotoxicity within the Que concentrations up to 2.5 μM over 24 h of cultivation (Supplementary Figure 1), supporting it was a safe and compatible therapeutic candidate for COPD. The cellular uptake of Que-lipo was observed using CLSM. The DiI labeling facilitated the observation of liposomes dispersion within the cell. The results in Figure 1E indicated that Que-lipo was efficiently internalized into A549 cells and accumulated in the cytoplasm. This was attributed to the liposomal formulation, which improved the cellular permeability and retention effect, thereby promoting better drug internalization. The CLSM in Figure 1E displayed significant uptake and distribution on A549 cells. This may be attributed to Que-lipo entered cells through endocytosis, and following release from the endosomes, which had been suggested that liposomes mainly rely on membrane fusion to escape the endosome (Gandek et al., 2023; Xu et al., 2021). These processes were crucial for the functionality of liposomes as a drug delivery system. The fluorescent intensity demonstrated Que-lipo mainly accumulated in the cytoplasm over 2 h, which provided a convincing guarantee for Que-lipo to adequately play their therapeutic role in the following *in vivo* applications.

In the COPD mouse model, the respiratory function parameters Penh, Te, and F revealed significantly elevated compared to control group (*p* < 0.001), as illustrated in Figure 2A. These parameters were critical indicators of respiratory mechanics and airway responsiveness, providing a comprehensive assessment of lung function. The significant increase in Penh indicated a heightened pause at the end of inspiration, which was associated with increased airway resistance and dynamic hyperinflation, a hallmark of COPD. The prolonged Te and increased F suggested alterations in expiratory flow and breathing frequency, respectively, which were indicative of the impaired gas exchange and ventilatory inefficiency commonly associated with COPD. These results demonstrated significant alterations in murine lung physiology, highlighting the model’s fidelity to the pathophysiological characteristics of COPD. Histological examination via H&E staining in Figure 2B revealed pronounced alterations in alveolar architecture along with the presence of inflammatory cell infiltration. This suggested that the structural changes in the lung tissue, such as alveolar wall destruction and inflammation, which were consistent with the pathologic features of COPD. Furthermore, masson staining and the quantification of fibrosis-associated protein expression presented. After masson staining, Figure 2C demonstrated that pulmonary fibrosis was observed under an optical microscope. Futhermore, quantification of TGF-β1 and α-SMA in model group exhibited significant elevation compared to normal group (Figure 2D). These findings were in line with the pathologic changes observed in COPD, where the destruction of lung tissue leaded to airspace enlargement and fibrosis. This not only confirmed the reliability of COPD model but also established a solid foundation for assessing the therapeutic potential of Que-lipo.

The i.t. method offers a non-invasive approach for directly delivering drugs into the lungs, making it especially beneficial for treating lung diseases like COPD. In contrast, i.p. injection provides a systemic route for drug administration. In this study, Que-lipo (0.3 mg/kg body weight of Que) was administered via both intratracheal and intraperitoneal routes to evaluate its therapeutic effects on COPD. Concurrently, a dose of 50 mg/kg of free Que was administered intraperitoneally, which had demonstrated effective therapeutic efficacy in treating pulmonary diseases. This design not only facilitates the exploration of Que-lipo’s potential in treating COPD but also enables the assessment of the significance of various administration routes in the treatment of pulmonary diseases. Que-lipo (i.t.) treatment positively improved various indexes compared to Que (i.p.) and Que-lipo (i.p.), including Penh, Te and F, which suggested that it may have a noticeable therapeutic impact on individuals with moderate to advanced stages of COPD (Figure 3A). While Que-lipo (i.p.) and Que (i.p.) demonstrated some enhancement in lung function, the overall improvement in detection indices was not as significant as that observed with Que-lipo (i.t.). After 30 days of treatment, H&E staining and score (Figure 3B; Table 1) revealed that Que-lipo (i.t.) enhanced alveolar uniformity and strengthens the integrity of alveolar wall structures, while also reducing the infiltration of inflammatory cells. Furthermore, masson staining and fibrosis score was performed in Masson trichrome–stained sections (Figure 3C; Table 1). Compared with the model group, the Que-lipo (i.t.) showed lower histopathological score and masson score, which was lower than Que (i.p.) and Que-lipo (i.p.). It indicated that i.t. administration of Que-lipo more effectively achieved a significant decrease in the fibrotic area. This may be attributed to the intratracheal administration of Que-lipo involved spraying medium into the lungs via nebulization. It resulted in the predominant deposition of Que-lipo in the lungs, while a minor portion is absorbed and subsequently transported to the liver and spleen (Supplementary Figure 2). These findings collectively supported i.t. of Que-lipo as a superior approach for enhancing lung function in mice with COPD.

Oxidative stress and inflammatory responses are concomitant symptoms of COPD (Barnes, 2020; Rao et al., 2020). To explore the Que-related treatment on resistance to oxidative stress, we investigated four parameters related to the oxidative status that include GSH-Px, SOD, MDA and MPO. A comprehensive evaluation oxidative stress status can be achieved by quantifying the concentrations of these biomarkers, which offered critical insights for the management of COPD treatment. In COPD mouse lung tissue, the reduced expression levels of GSH-Px and SOD in Figure 4A reflected a weakened antioxidant capacity. This was an important marker of the oxidative stress state in COPD. After 30-day treatment with Que and Que-lipo, there was an observed increase in GSH-Px and SOD levels, along with a concomitant decrease in MDA and MPO levels (Figure 4A). And Que-lipo (i.t.) showed the greatest reduction in oxidative stress compared with Que-lipo (i.p.) and Que (i.p.). The findings from inflammatory factor detection further supported Que-lipo’s therapeutic potential for COPD and highlighted the advantages of the i.t. route in suppressing COPD-related inflammatory responses.

Inflammatory responses and oxidative stress often coexist or trigger each other in COPD, forming a vicious cycle and deteriorating the lung tissue damage. The model group presented distinct features of COPD, characterized by notably increased levels of IL-6 and TNF-α (Figure 4B). The quantitative analysis of these cytokines in Figure 4D showed they were significantly higher compared to the control group (*p* < 0.001). While IL-10, an anti-inflammatory cytokine, exhibited a contrasting pattern in Figures 4C,E, suggesting a shift towards a more pro-inflammatory state in the model group. Que-related treatments resulted in pronounced reductions in IL-6 and TNF-α, as well as increases in IL-10, suggesting a potent suppression of inflammation, which was critical to alleviating the inflammatory burden in COPD. These results underline the anti-inflammatory potential of Que and Que-lipo. Moreover, further quantitative analysis of the above results revealed the best anti-inflammatory effect of Que-lipo via i.t. (Figures 4D,E). The treatment ability of Que-lipo to modulate the cytokine profile towards a more anti-inflammatory state is a significant therapeutic advantage, as it addresses the underlying inflammation that drives COPD progression. The preferential efficacy of Que-lipo when administered intratracheally highlights the importance of route of administration in maximizing the therapeutic benefits, likely due to the direct delivery of the treatment to the affected lung tissues. These findings collectively contribute to a deeper understanding of the mechanisms by which Que-lipo exerts its anti-inflammatory effects and provide a scientific basis for its potential clinical application in COPD treatment.

Dysregulation of apoptosis likely plays a crucial role in the pathological progression of COPD. Caspase-dependent apoptosis is a key pathway in cell death (Tajbakhsh et al., 2020). Assessing biomarkers of apoptotic responses can provide a comprehensive understanding during the treatment of COPD. Our findings revealed reduced Bcl-2 levels and increased caspase-3/7 levels in mice with COPD (Figure 5A), indicating heightened apoptotic activity. The decreased staining intensity of Bcl-2 and the increased staining intensity of caspase-3/7 in model group confirmed the important role of apoptosis in COPD. After treatment, the enhanced treatment outcomes were observed in all Que-related groups, which can be exemplified by the intensity of immunohistochemical staining. This suggested that Que can inhibit apoptosis by regulating the expression of these apoptosis-related proteins, thereby slowing the pathological progression of COPD. These results were further verified by the quantitative analysis of IOD. As depicted in Figure 5C, the significant differences in IOD of Bcl-2 and caspase-3/7 were found between the model and control group (*p* < 0.001). Notably, the Que-lipo (i.t.) group exhibited the most significant anti-apoptotic effects compared to Que (i.p.) and Que-lipo (i.p.) after treatments. This suggested that Que-lipo can inhibit apoptosis by regulating the expression of these apoptosis-related proteins, thereby slowing the pathological progression of COPD. To further investigate the effects of Que-lipo in inhibiting apoptosis associated with COPD, similar strategies were utilized to identify apoptotic cells in the lungs through TUNEL staining. Figure 5B indicated that the number of positive cells reduced noticeably in Que-related treatment groups. The noticeable reduction in the number of positive cells (Figure 5B) and the markedly decreased apoptosis ratio (Figure 5C) demonstrated that Que-related treatment effectively reduced apoptosis in COPD mice. The significant difference judged by confidence levels of *p* < 0.05 (indicated by @) demonstrated that the optimal anti-apoptosis effect of Que-lipo (i.t.), which allowed Que-lipo for more direct action on the lungs.

While Que had shown promise in treating COPD, the precise mechanisms underlying its efficacy remain incompletely understood. This study analyzed the key component’s expression levels of the lung tissue inflammasome by immunohistochemistry and RT-PCR, including caspase-1, IL-1β and NLRP3. Additionally, the potential impact of Que (i.p.), Que-lipo (i.p.) and Que-lipo (i.t.) on these inflammatory markers was examined. Following 30 days treatment, the results exhibited in Figure 6A showed that the levels of caspase-1 and IL-1β significantly decreased, which indicated the inflammation conditions were improved considerably. Based on these preliminary insights, we further quantified the immunohistochemical proteins caspase-1 and IL-1β by average IOD. As shown in Figure 6B, all treatment groups exhibited a significant downregulation of these markers (*p* < 0.05). Among them, Que-lipo (i.t.) had the best effect. Further, Figure 6C indicated that the trends of changes in the mRNA levels of NLRP3 were the same to those of inflammatory markers. Following treatment, the obvious markedly decreased levels of inflammatory factors in Que (i.p.), Que-lipo (i.p.) and Que-lipo (i.t.), which suggested a noteworthy therapeutic response. The similar trends in NLRP3 mRNA expression suggested that Que might mitigate airway inflammation in COPD mice by regulating the NLRP3-(caspase-1)/IL-1β inflammatory signaling pathway.

As COPD progresses, persistent inflammatory responses and damaged tissue can lead to fibrotic lesions in the lungs. In this study, we employed RT-PCR analysis to examine the expression of fibrosis-related signaling molecules, including TGF-β1, Rho and ROCK. These biomarkers played a role in the pathophysiological process of COPD, including promoting inflammatory responses, airway remodeling and fibrosis. The mRNA levels of these proteins presented in Figure 7 revealed a significant increase in model group compared to the control group, indicating characteristic pathological changes in COPD. Following treatment with Que and Que-lipo, the mRNA expression levels of TGF-β1, Rho and ROCK were significantly decreased, especially in Que-lipo (i.t.) group. The Que-lipo via intratracheal administration decreased the mRNA levels of TGF-β1, Rho and ROCK to approximately normal levels. These findings suggested that transtracheal delivery of Que-lipo can effectively alleviate fibrotic symptoms in a murine model of COPD.

Previous research had confirmed that TGF-β1 protein involved Smad signaling induce the expression of α-SMA in lung fibroblasts (Ji et al., 2014). And the Rho/ROCK signaling pathway likely contributed in a supportive or synergistic manner during this biological process. This observation was consistent with our experimental findings regarding the changes in mRNA levels. It suggested that Que-lipo may exert a significant inhibitory effect on fibrosis progression in COPD mice by modulating the expression of these crucial signaling molecules. Considering all factors, Que-lipo showed significant therapeutic promise in a murine model of COPD by effectively regulating inflammatory responses and inhibiting the fibrotic process. The anti-inflammatory and anti-fibrotic properties of Que-lipo likely stem from its modulation of the NLRP3/IL-1β inflammasome pathway and the TGF-β fibrosis-related signaling pathway. This finding opens new avenues for therapeutic strategies in COPD management. Future research will investigate the potential clinical benefits of Que-lipo to offer safer and more effective treatment options for COPD patients.

## 5 Conclusion

In summary, we successfully developed Que-lipo, which not only improved the solubility and biocompatibility of Que but also demonstrated effective cellular uptake *in vitro*. Administered via pulmonary delivery, Que-lipo regulated the expression of key apoptosis-associated proteins such as Bcl-2 and caspase-3/7, leading to significant inhibition of apoptotic activity in COPD. Furthermore, Que-lipo markedly enhanced its ability to alleviate lung inflammation and fibrosis symptoms by modulating inflammation-related factors and fibrosis signaling molecules. The potential mechanisms of Que-lipo in treating COPD were elucidated, including the suppression of the NLRP3/IL-1β inflammasome pathway and the TGF-β1-related fibrosis signaling pathway. Future studies will further explore the clinical implications of Que-lipo, aiming to provide improved and safer therapeutic options for individuals with COPD.

## Data Availability

The original contributions presented in the study are included in the article/Supplementary Material, further inquiries can be directed to the corresponding authors.
