# Supplementary material for: Mitigating inflammation and fibrosis: the therapeutic potential of quercetin liposomes in COPD
Source: Front Pharmacol. 2024 Dec 17;15:1503283. doi: 10.3389/fphar.2024.1503283 (PMC11685140; doi:10.3389/fphar.2024.1503283)
Supplement: Supplementary file 1 [file DataSheet1.docx]

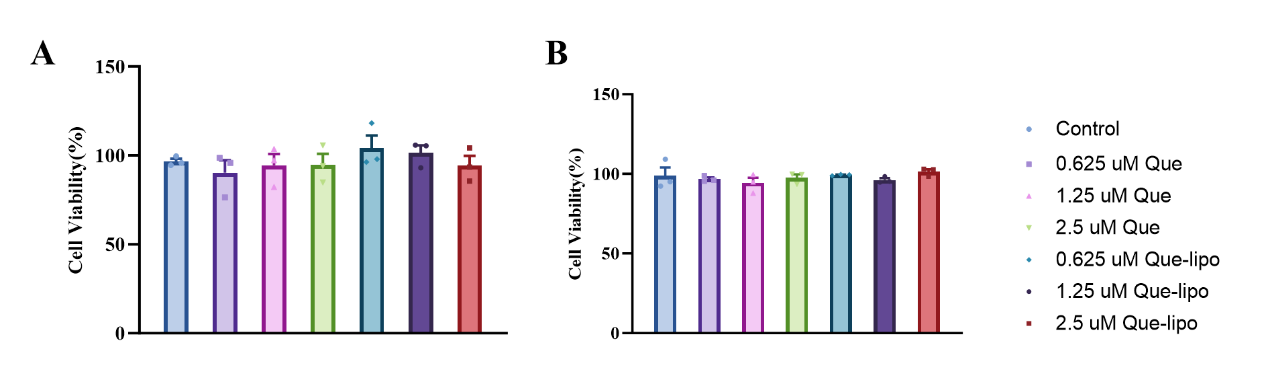


**Fig. S1**. In vitro cytotoxicity of Que and Que-lipo on A549 and Beas-2B cells.


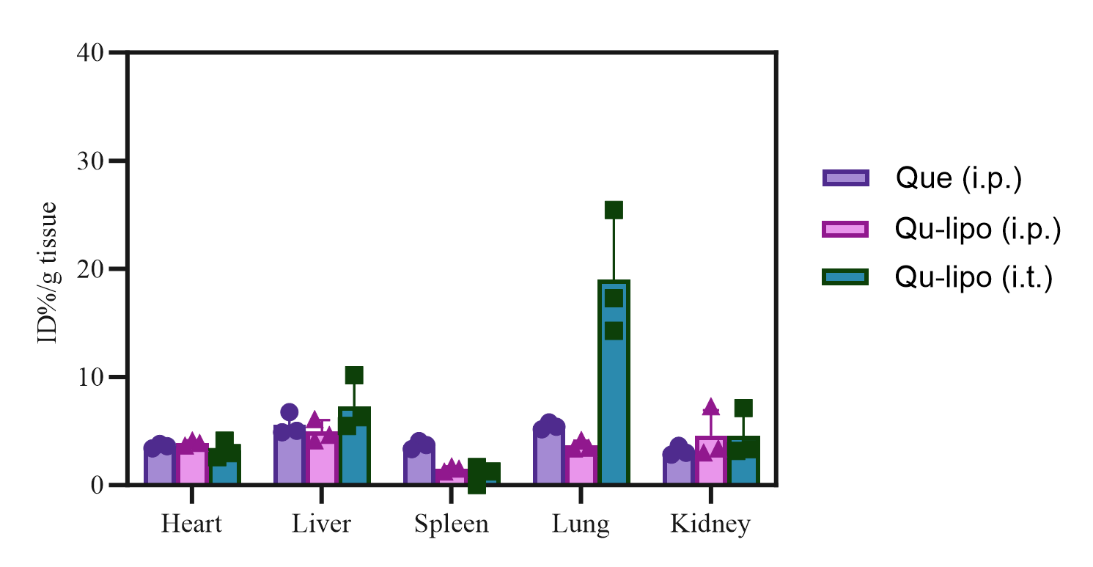


**Fig. S2**. Organ distribution of Que expressed as % ID/g of tissue at 24 h post-administration. Data represent mean ± SD (n = 3).
